# Temporal and spatial distribution of polio vaccine coverage in Brazil between 1997 and 2021

**DOI:** 10.1590/1980-549720230037

**Published:** 2023-08-28

**Authors:** Nathanael de Souza Maciel, Hévila Medeiros Ferreira Gomes Braga, Francisca Jessika Nunes de Moura, Francisco Jardsom Moura Luzia, Isabelle e Silva Sousa, Emilia Soares Chaves Rouberte

**Affiliations:** I Universidade Estadual do Ceará, Health Sciences Center – Fortaleza (CE), Brazil.; II Universidade da Integração Internacional da Lusofonia Afro-Brasileira, Health Sciences Institute – Redenção (CE), Brazil.

**Keywords:** Poliomyelitis, Poliovirus vaccines, Vaccines, Time series studies, Public health, Geographic mapping, Poliomielite, Vacinas contra poliovírus, Vacinas, Estudos de séries temporais, Saúde pública, Mapeamento geográfico

## Abstract

**Objective:**

To analyze the temporal and spatial distribution of polio vaccine coverage in Brazilian states.

**Methods:**

An ecological time series study was conducted using data from the National Immunization Program Information System. The analyzed period was from 1997 to 2021. Joinpoint software was used to calculate the annual percentage change and average annual percentage change through regressions. QGIS 3.10.7 software was used to construct thematic maps. GeoDa 1.20.0.10 software was used to estimate spatial autocorrelation using the Global Moran's Index and Local Moran's Index.

**Results:**

National vaccine coverage in 1997 was 89.27%, decreasing to 61.32% in 2021. The trend analysis indicated an average annual decrease of 1.5% in polio vaccine coverage in Brazil. Across the country, 17 states showed a statistically significant reduction in the average annual percentage change rate. The highest average reduction rates in vaccine coverage among Brazilian states were observed in Amapá (−3.7%; 95%CI −6.0; −1.4) and Pernambuco (−3.3%; 95%CI −4.0; −2.5). In the spatial analysis, in Moran Global, a positive autocorrelation was identified in the years 2012 to 2021 (p<0.02), with an index value of 0.361, which means that geographically close areas tended to have similar levels of vaccination coverage.

**Conclusion:**

There was significant heterogeneity in coverage among states and a strong decrease trend in vaccination rates, which could facilitate the circulation of the poliovirus and pose a threat to the susceptible population.

## RESUMO

**Objetivo:**

Analisar a distribuição temporal e espacial da cobertura da vacina contra poliomielite nos estados brasileiros.

**Métodos:**

Estudo ecológico de séries temporais, cuja fonte de dados foi o Sistema de Informação do Programa Nacional de Imunizações. O período analisado foi de 1997 a 2021. Utilizou-se o *software* Joinpoint para calcular a variação percentual anual e variação percentual anual média por meio de regressões. Para construção de mapas temáticos foi utilizado o *software* QGis 3.10.7. Para estimar a autocorrelação espacial com o Índice de Moran Global e Índice de Moran Local foi utilizado o *software* GeoDa 1.20.0.10.

**Resultados:**

A cobertura vacinal nacional em 1997 foi de 89,27%, passando para 61,32% em 2021. A análise de tendência apontou o decréscimo médio de 1,5% ao ano na cobertura da vacina contra poliomielite no Brasil. Em todo o país, 17 estados apresentaram redução estatisticamente significativa na taxa de variação percentual anual média. As maiores taxas médias de redução da cobertura vacinal entre os estados brasileiros foram observadas no Amapá (−3,7%; IC95% −6,0; −1,4) e em Pernambuco (−3,3%; IC95% −4,0; −2,5). Na análise espacial, no Moran Global, foi identificada autocorrelação positiva nos anos de 2012 a 2021 (p<0,02), com valor de índice de 0,361, o que significa que as áreas geograficamente próximas tenderam a ter níveis semelhantes de cobertura vacinal.

**Conclusão:**

Evidenciou-se expressiva heterogeneidade na cobertura entre os estados e forte tendência de queda dos índices, o que pode propiciar a circulação do poliovírus e colocar sob ameaça a população susceptível.

## INTRODUCTION

Poliomyelitis is an infectious-contagious viral disease of significant clinical importance due to the high rate of infectivity and the lack of specific treatment that enables the clinical cure of infected individuals^1^. The clinical particularities of the disease manifest themselves widely among those infected and are directly related to the impairment and dissemination of the virus in the nervous system, ranging from asymptomatic forms to severe clinical forms of irreversible paralysis, respiratory failure, and death^1,2^.

Due to the characteristics of transmission, poliomyelitis has an endemicity linked to the conditions of basic sanitation and development of a country or region. Thus, emerging countries faced similar trajectories in relation to the magnitude of epidemics and morbidity and mortality conditions of the population^3^.

Endemicity patterns only changed from the 1950s onward, when safe and effective vaccines were developed, making it possible to implement a disease prevention and control network. However, vaccination strategies were different between countries, which meant that the disease was completely disseminated in some regions and remained endemic in countries where access to immunobiologicals was compromised due to financial and geographic reasons^4,5^.

Vaccination coverage rate is a powerful health indicator, which allows identifying aspects of child health and the effectiveness of health services and contributes to the planning of actions in this area^6^. The Pan American Health Organization has established achieving immunization coverage levels of 95% or greater as a primary goal for poliomyelitis in the Americas, with a view to controlling vaccine-preventable diseases and maintaining polio eradication on the continent^7^.

In Brazil, the epidemiological path to eliminate poliomyelitis was surrounded by successes and obstacles^2^. With the onset of the National Immunization Program (*Programa Nacional de Imunizações* – PNI) in 1973, and the beginning of vaccination campaigns against poliomyelitis (child paralysis) in 1980, the country began to interrupt the dissemination cycle of the virus, being considered free of the disease in 1990^3^.

The elimination of the disease by the wild virus was exclusively due to the oral vaccine of attenuated viruses, based on the inclusion of the oral polio vaccine (OPV) vaccine schedule and later the inactivated polio vaccine (IPV), introduced in the schedule in 2011 to prevent rare adverse events caused by OPV^8^, such as vaccine-associated poliomyelitis and national vaccination campaigns. Since 2016, the vaccination schedule against poliomyelitis consists of three doses of IPV at two, four, and six months and two boosters with OPV at 15 months and four years of age^9^.

Despite the advances promoted by vaccination, in recent years there has been a decline in adherence to the vaccination schedule and in the coverage of oral and inactivated vaccines against poliomyelitis. In addition, there was a setback in the country's income distribution, highlighting issues of child vulnerability and access to favorable sanitary conditions^10^. Another factor causing this decrease in adherence is the hesitation or reluctance of those responsible for taking children to be vaccinated, despite the availability of the vaccine in public health institutions. Such factors can threaten the advances provided by immunization^10^. It is emphasized that the drop in vaccination coverage in the child population in Brazil is a worrying problem that is not limited to a single vaccine.

Based on an overview of the spatial and temporal distribution rates of vaccination coverage, it is possible to identify oscillations, enabling the adoption of strategies that favor the implementation of the complete vaccination schedule. In the case of OPV and IPV, the identification also favors the prevention of new cases of the disease and the recognition of regions with a higher risk of dissemination. Therefore, the objective was to analyze the temporal and spatial distribution of polio vaccine coverage in the Brazilian states.

## METHODS

This is an ecological study of time series, in which vaccination coverage against poliomyelitis was analyzed in the 27 states of the federation. Data collection took place in November and December 2022.

The electronic portal of the Department of Informatics of the Unified Health System (*Departamento de Informática do Sistema Único de Saúde* – DataSUS) was used to obtain the data. Subsequently, data on health care were sought and, thus, the National Immunization Program Information System (*sistema de Informação do Programa Nacional de Imunização* – SI-PNI) was selected.

Data regarding vaccination coverage were selected according to year and states, regions and total. As a filter, we used the selection of immunobiologicals for poliomyelitis, poliomyelitis (1^st^ booster) and poliomyelitis (four years). It is emphasized that the vaccination coverage was extracted already calculated from the information system, where the formula considers the number of applied doses of the indicated dose (first, second, third dose or single dose, depending on the vaccine) divided by the target population, multiplied by 100^11^.

Although data have been available since 1994, the period analyzed by the authors was from 1997 to 2021, due to the amount of missing information in the database. The final date was set due to being the last year with complete data. The data available online was downloaded to CVS format.

Raw data were organized in a Microsoft Office Excel spreadsheet. Subsequently, they were organized into tables in Microsoft Excel^®^ and imported into the Joinpoint Regression Program^®^ version 4.6.0.0. Using this software, a segmented linear analysis was carried out (analysis by inflection points or joinpoints), performing the logarithmic transformation of the values^12^.

The annual percentage change (APC) was calculated, with a 95% confidence interval (95%CI), in which a negative APC value indicates a decreasing trend, and a positive value indicates an increasing trend^13^. Average Annual Percentage Change (AAPC) was calculated, which shows how change occurred over the entire period. The model was adjusted so that the number of inflection points, that is, the amount of changes in the linear trend that could be generated, could vary from zero to two, that is, up to three segments. Results with p<0.05 were considered significant.

For the construction of thematic maps, an administrative-political map with state divisions of Brazil (in shapefile format — shp.) extracted from the Brazilian Institute of Geography and Statistics (*Instituto Brasileiro de Geografia e Estatística –* IBGE) was used. With the use of the QGis 3.10.7 software, maps were constructed with the time frames delimited in the regression stage. Thematic maps correspond to the average vaccination coverage of each state in the periods 1997–2005, 2006–2013, 2014–2021 and total, 1997–2021. These tables were based on the inflection points of the temporal trend for Brazil, which were identified in previous analyzes with the data obtained in this study. It should be noted that the vaccination coverage recommended by the PNI is 95%.

For spatial dependence analysis, the neighborhood matrix was constructed considering the nearest direct neighbor, of the first order queen type. Spatial autocorrelation was estimated with the Global Moran Index, with 99 permutations, and the presence of clusters and outliers using the Local Moran Index (LISA), using the scatter plot of the Moran Index, significance map and LISA cluster map. GeoDa 1.20.0.10 was used for this purpose.

In the Global Moran Index, values close to 0 indicate absence of spatial autocorrelation, and the closer the variation is to −1 and +1, it indicates negative or positive correlation. LISA allows evaluating the occurrence of cluster-type spatial clusters, identifying the distribution of vaccination coverage in the municipality in relation to its close neighbors, considering only those with statistical significance (p<0.05), so that it can be of the type: High-High (HH), municipalities with high vaccination coverage rates close to municipalities with high rates; Low-Low (LL), municipalities with low rates close to low ones; Low-High (LH), municipalities with low rates close to high; High-Low (HL), municipalities with high rates close to low^14^.

This study is a research with secondary and published data, therefore it did not require submission and approval by the ethics committee for research with human beings.

## RESULTS

**Figure 1:**
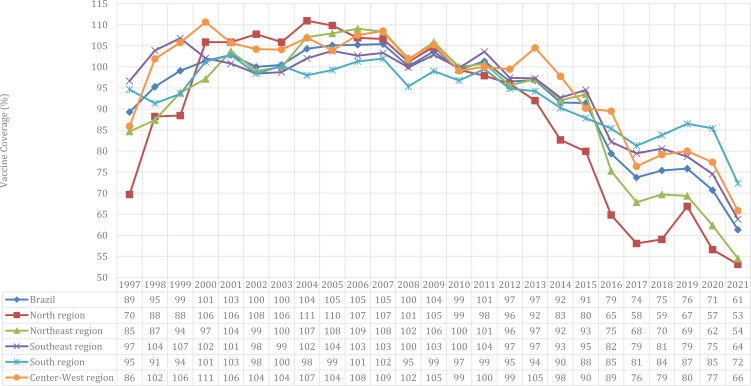
Polio vaccine coverage, according to Brazilian regions. Brazil, 1997–2021.

Considering the period from 1997 to 2021, the average vaccination coverage in Brazil was 93.08%. In 1997, national coverage was 89.27%, and then 61.32% in 2021. Regarding the Brazilian regions, the Southeast and Northeast stood out, reducing from 96.64 and 84.65% in 1997 to 63.80 and 54.47% in 2021, respectively. Data regarding polio vaccination coverage according to the regions of Brazil are presented in Figure 1.

During the study period, the states with the highest average coverage were Espírito Santo (101.92%) and Pernambuco (100.13%). The states with the lowest coverage were Amapá (83.06%) and Amazonas (84.86%). Table 1 shows the annual percentage change in polio vaccine coverage.

**Table 1:** Annual percentage variation in polio vaccine coverage, by federative unit. Brazil, 1997 to 2021.

Local	APC1 (95%CI)	IP	APC2 (95%CI)	IP	APC3 (95%CI)	AAPC (95%CI)
Brazil	1.6* (0.4; 2.9)	2005	−1.2 (−2.7; 0.3)	2013	−5.0* (−6.1; −3.8)	−1.5* (−2.2; −0.8)
North	14.5* (3.9; 26.2)	2000	−0.3 (−2.1; 1.5)	2010	−6.1* (−7.3; −4.9)	−1.3 (−2.7; 0.1)
Acre	10.9* (3.0; 19.3)	2002	−1.9 (−5.3; 1.7)	2011	−6.8* (−9.2; −4.4)	−1.5 (−3.6; 0.6)
Amapá	1.8 (−1.7; 5.5)	2009	−9.0* (−12.2; −5.7)			−3.7* (−6.0; −1.4)
Amazonas	0.2 (−0.7; 1.1)	2013	−5.2* (−7.5; −2.8)			−1.6* (−2.6; −0.7)
Pará	28.5* (8.8; 51.8)	2000	−0.2 (−3.8; 3.4)	2009	−8.0* (−9.8; −6.1)	−1.1 (−3.5; 1.4)
Rondônia	1.7* (0.5; 2.8)	2009	−3.8* (−4.9; −2.8)			−1.1* (−0.4; −3.0)
Roraima	−0.4 (−1.3; 0.5)	2017	−12.9* (−21.5; −3.5)			−2.6* (−4.3; −0.9)
Tocantins	0.2 (−0.8; 1.2)	2007	−2.9* (−3.4; −2.3)			−1.6* (−2.1; −1.1)
Northeast	3.0* (1.4; 4.5)	2005	−1.5 (−3.3; 0.3)	2013	−6.5* (−7.0; −5.1)	−1.8* (−2.6; −0.9)
Alagoas	24.2* (12.0; 37.7)	2000	0.5 (−1.4; 2.4)	2010	−4.0* (−5.4; −2.7)	1.0 (−0.5; 2.6)
Bahia	10.6* (3.1; 18.7)	2001	−0.7 (−2.2; 0.8)	2013	−7.9* (−10.1; −5.6)	−1.4 (−2.9; 0.1)
Ceará	0.0 (−0.6; 0.6)	2015	−7.1* (−10.1; −4.1)			−1.8* (−2.7; −1.0)
Maranhão	8.6* (6.1; 11.2)	2005	−2.7 (−5.5; 0.1)	2013	9.1* (−11.2; −7.0)	−1.3* (−2.7; 0.0)
Paraíba	2.0* (0.9; 3.2)	2009	−4.8* (−5.9; −3.7)			−1.5* (−2.2; −0.7)
Pernambuco	−1.4* (−1.9; −0.9)	2015	−8.6* (−11.1; −6.0)			−3.3* (−4.0; −2.5)
Piauí	24.6* (6.0; 46.4)	1999	1.2 (−1.0; 3.4)	2007	−4.3* (−5.0; −3.5)	−0.3 (−1.8; 1.2)
Rio Grande do Norte	3.6* (1.6; 5.7)	2007	−4.1* (−5.3; −3.00			−1.0 (−2.0; 0.0)
Sergipe	0.0 (−0.6; 0.7)	2012	−5.8* (−7.0; −4.50			−2.2* (−2.8; −1.6)
Southeast	0.0 (−0.4; 0.5)	2012	−4.2* (−5.2; −3.3)			−1.6* (−2.0; −1.2)
Espírito Santo	10.3 (−4.1; 26.8)	1999	−1.5* (−2.3; −0.7)	2012	−4.0* (−5.2; −2.7)	−1.5* (−2.7; −0.3)
Minas Gerais	8.8* (0.2; 18.0)	2000	0.5 (−0.8; 1.8)	2011	−3.3* (−4.5; −2.1)	−0.1 (−1.3; 1.1)
Rio de Janeiro	1.0* (0.0; 2.0)	2010	−5.3* (−7.6; −2.9)	2018	−15.2* (−22.7; −6.9)	−3.3* (−4.6; −1.9)
São Paulo	−0.7* (−1.2;-0.2)	2013	−3.3* (−4.7; −1.8)			−1.6* (−2.1; −1.0)
South	0.8 (−0.1; 1.70	2007	−1.8* (−2.4; −1.3)			−0.7* (−1.2; −0.3)
Paraná	0.1 (−0.5; 0.7)	2011	−2.8* (−3.8; −1.9)			−1.1* (−1.6; −0.6)
Rio Grande do Sul	0.8 (−0.3; 1.9)	2007	−1.9* (−2.5; −1.3)			−0.8* (−1.3; −0.2)
Santa Catarina	1.0* (0.1; 1.9)	2007	−1.4* (−2.0; 0.9)			−0.4 (−0.9; 0.0)
Center-West	11.1 (−0.9; 24.7)	1999	−0.5 (−1.1; 0.1)	2013	−4.6* (−5.8; −3.4)	−1.0 (−2.0; 0.0)
Distrito Federal	−1.5* (−2.2; −0.9)					−1.5* (−2.2; −0.9)
Goiás	21.3* (4.6; 40.6)	1999	0.2 (−0.8; 1.2)	2011	−4.8* (−5.9; −3.7)	−0.3 (−1.6; 1.0)
Mato Grosso	4.5 (−1.9; 11.3)	2000	−1.4* (−2.2; −0.7)	2013	−4.4* (−5.7; −3.0)	−1.7* (−2.6; −0.8)
Mato Grosso do Sul	10.4 (−2.5; 25.0)	2000	−0.7 (−1.5; 0.1)	2019	−16.3 (−34.7; 7.2)	−0.8 (−3.2; 1.7)

APC: annual percentage change; 95% CI: 95% confidence interval; IP: inflection point; AAPC: average annual percentage change;

* statistically significant (p<0.05).

Trend analysis pointed to the mean yearly decrease in polio vaccine coverage in Brazil (AAPC: 1.5%; 95%CI −2.2; −0.8). With the exception of the North (AAPC: −1.3; 95%CI −2.7; 0.1), the regions of Brazil showed a statistically significant average annual decrease, with emphasis on the Northeast (AAPC: −1.8%; 95%CI 2.6; −0.9) and the Southeast (AAPC: −1.6%; 95%CI −2.0; −1.2). The lowest average annual decrease was identified in the South region (AAPC: −0.7%; 95%CI −1.2; −0.3).

Nationwide, 17 states showed a statistically significant reduction in the average annual percentage change rate of polio immunization coverage. Ten states did not show statistical significance, being considered stationary. The highest average rates of reduction in vaccination coverage among Brazilian states were seen in Amapá (AAPC: −3.7%; 95%CI −6.0; −1.4) and Pernambuco (AAPC: −3.3%; 95%CI −4.0; −2.5).

In the North region, the states of Acre and Pará did not have a statistically significant reduction. Amapá and Rondônia had the highest average annual reduction (AAPC: −3.7 and −3.3%; 95%CI −6.0; −1.4 and −0.4; −3.0, respectively). In the Northeast region, Pernambuco had a reduction (AAPC: −3.3%; 95%CI −4.0; −2.5), followed by Ceará (AAPC: −1.8%; 95%CI −2.7; 0.0). In the Southeast region, Rio de Janeiro had the highest percentage change (AAPC: −3.3%; 95%CI −4.6; −1.9). It should be noted that, for this state, there was a turning point in 2018, with a statistically significant reduction (APC: −15.2%; 95%CI −22.7; −6.9). In the South region, the biggest change occurred in Paraná (AAPC: −1.1%; 95%CI −1.6; −0.6). In the Center-West, the state of Mato Grosso had the greatest change (AAPC: −1.7; 95%CI −2.6; −0.8). In this region, Mato Grosso do Sul stands out, where 2019 was the turning point for change per year (APC: −16.3%; 95%CI −34.7; 7.2), although statistical significance is not shown, according to the data presented in Table 1.

**Figure 2:**
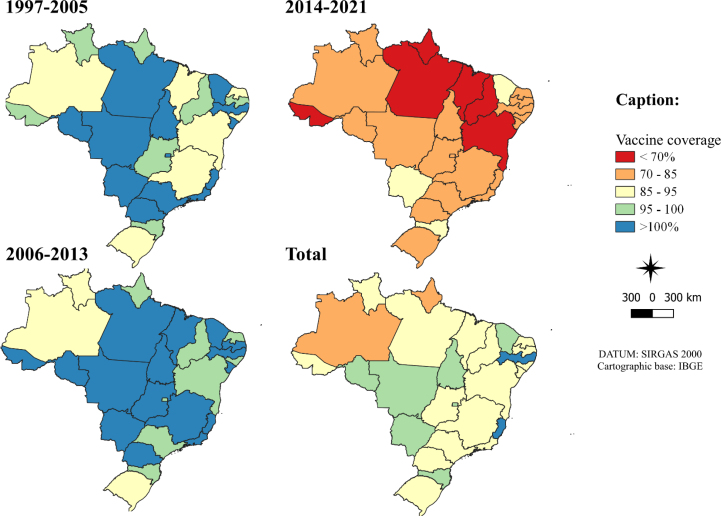
Temporal and spatial distribution of polio vaccine coverage, according to states. Brazil, 1997 to 2005, 2006 to 2013, 2014 to 2021, and 1997 to 2021.

According to the regions of the country in the analysis from 1997 to 2021, only the Center-West region had average coverage for the polio vaccine greater than 95% (96.46%), with only the state of Goiás having lower coverage (93.84%). The region with the lowest coverage was the North (88.73%), with emphasis on coverage in the states of Amapá (83.06%) and Amazonas (84.86%). Data regarding polio vaccination coverage by period in the Brazilian states are presented in Figure 2.

In the first period, from 1997 to 2005, all regions of the country had vaccination coverage greater than 97.73%. As for the states, seven had lower coverage than recommended, 95%, ranging from 87.1% (Alagoas) to 94.24% (Rio Grande do Sul). The highest vaccination coverage recorded was in Espírito Santo (116.04%) and, with it, 13 states had coverage greater than 100%.

In the period from 2006 to 2013, all regions of the country continued with vaccination coverage greater than 97.87%. The states of Amazonas (90.66%), Roraima (92.79%), and Rio Grande do Sul (94.24%) were the only ones with coverage below the established goal. In total, 16 states had vaccination coverage greater than 100%, with Rondônia standing out (109.16%).

In the period from 2014 to 2021, there was a drastic change in terms of coverage. The biggest regional drop occurred in the North (65.12%), with the others ranging from 73.06% (Northeast) to 84.1% (South). The state with the lowest recorded coverage was Amapá (51.34%) and the highest coverage was in Mato Grosso do Sul (93.23%).

In the analysis of the Global Moran Index, the only period with a significant pseudo-p (p<0.02) was from 2014 to 2021, with an index value of 0.361, indicating the existence of positive autocorrelation, with areas tending to be similar with regard to vaccination coverage.

In the LISA by period, the presence of clusters and outliers was noticed, through the combination of coverage between the analyzed state and those around it. Considering p<0.05, it was found that, from 1997 to 2005, there was a state of high coverage surrounded by low coverage (Sergipe); in the period from 2006 to 2013, no significant results were identified; from 2014 to 2021, there were three states with high coverage close to high (Paraná, São Paulo and Mato Grosso do Sul), one high close to low (Tocantins) and one low close to low (Amapá). Analyzing the time interval from 1997 to 2021, it was considered a low type close to low (Roraima).

## DISCUSSION

During the evaluated period, polio vaccine coverage was not homogeneous between Brazilian states and regions. It was observed that the North region, one of the regions with the worst social and economic indicators in the country^15^, had the lowest coverage rates. This finding can be justified by the extension of coverage and the geographic access barriers related to the concentration of Family Health teams in urban spaces, adding barriers to the arrival of users to the units, such as difficulties in welcoming spontaneous demand, scheduling appointments and availability of transport for care^16^.

The analysis points out that, among the regions of the country, only the Center-West, with the exception of the state of Goiás, managed to maintain an average coverage above 95% during the evaluated period. It is important to mention that the Center-West was one of the regions where the greatest increase in the availability of immunizations was observed, with the greatest tendency to increase the availability of vaccines in Goiás, in the period between 2012 and 2018.

Statistically significant reductions in polio vaccination coverage rates occurred in 17 states. These resulted from multiple causes, among which vaccine hesitancy has been gaining prominence, which consists of a complex event, characterized by refusal or delay in accepting vaccines made available by the health system. Associated with this phenomenon, there is the idea that vaccination is an unnecessary effort, especially with regard to vaccines that prevent less prevalent or previously eradicated diseases, as is the case of polio in Brazil^17,18^.

The existence of low vaccination coverage rates is worrying, since it increases the risk of reemergence and loss of control of diseases preventable by immunization. In some cases, lack of access to adequate health services, information about the benefits of vaccination and resistance from some groups contributed to suboptimal vaccination coverage for certain diseases^19^.

In addition, vaccination coverage in the Brazilian child population in relation to other diseases has shown some variations over time. Since the 1990s, childhood vaccination coverage was above 95%. However, in Brazil, the decline in vaccination coverage began in 2012, intensifying from 2016 onwards, being further aggravated by the COVID-19 pandemic^20^.

There are many factors that motivate this drop. The underfunding of the Brazilian Unified Health System (*Sistema Único de Saúde* – SUS), due, for example, to Constitutional Amendment 95 in 2016, may have had an impact on vaccination and vaccination coverage in Brazil, since the limitation of public spending imposed by this measure resulted in a freeze on resources allocated to health, which do not adequately accompany the increase in demands and needs of the system^21^. In addition to the weakening of SUS, there are technical issues such as the implementation of a new immunization information system and social and cultural factors that impact vaccination adherence^19,22,23^.

Distrust in the efficacy and safety of the vaccine also contributes to vaccine hesitancy^19^. In this regard, it reinforces the problem of fake news, subsidized by anti-vaccination movements, in reinforcing suspicions around immunizations in Brazil, having identified several false news that presented vaccines as ineffective or as potential threats to health^24^. Given this scenario, it should be noted that social networks are an important space nowadays for the search for scientific information, whose potential must be explored to promote participation and access to scientific knowledge^25^.

In addition to vaccine hesitancy, another factor that may have influenced the drop in polio vaccine coverage rates is the change in the PNI information systems. The system, which previously had a record of applied doses, now has a nominal record of vaccination, offering numerous benefits and possibilities for more detailed statistical monitoring. However, the transition period to the new system may have generated inconsistencies in vaccination records, given its complexity and the heterogeneity with which it was implemented in states and municipalities^26,27^.

It is important to mention that difficulties in accessing the internet, especially in more remote regions, such as the North and Northeast of Brazil, may have an influence on vaccination data. Adequate feeding of information systems on vaccination depends on an efficient system for recording and updating information, which can be compromised by lack of connectivity^28^.

In addition, it is worth mentioning that the pandemic scenario of COVID-19 caused interruptions in vaccine distribution services, negatively impacting the practice of immunizations in a timely manner^29^. However, the indices were already falling before the onset of the pandemic, which indicates that this may have contributed to its reduction, but it is not the only factor.

The COVID-19 pandemic brought additional challenges to the downward trend in vaccine coverage, with delays in vaccine administration and vaccine hesitancy^30^. In addition, the lack of immunizations is a relevant factor in the drop in vaccination coverage observed in the country in recent years and can cause damage to the reliability of the PNI, giving strength to the anti-vaccination movement^31^.

The decline in vaccination coverage in childhood, including poliomyelitis, has been particularly relevant in the last decade in Brazil. During the period from 2011 to 2021, a study identified that the biggest drops in vaccination coverage were identified in states and health regions with greater social vulnerability, especially in the North and Northeast states, which was accentuated during the pandemic^30^.

On the other hand, in the period from 1997 to 2013, several states had vaccination coverage above 100%, a fact that raises reflection on the actual scope of coverage and is possibly related to the form of registration of vaccines, the revaccination of children or the care of children belonging to neighboring states. In this scenario, Primary Health Care plays a fundamental role due to its greater proximity to the population and the commonly established relationship of trust. In order to obtain vaccination coverage closer to the expected, it is up to health professionals to search for children with pending vaccinations, through the evaluation and systematic review of vaccination cards^32^.

In addition to the active search for unvaccinated people in the target populations, strategies should include dissemination in traditional and electronic media, partnerships with schools and universities, expansion of opening hours at vaccination stations, mobilization of civil society and collaboration of scientific societies in partnership with the three management instances, as well as the establishment of intra and intersectoral partnerships^33^.

It is noteworthy that evaluating the spatial disparities of the country's regions is important to understand the distribution of health indicators and to identify areas with low vaccination coverage^34^, since the identification of these areas helps to prevent epidemics of already controlled diseases and to promote vaccination strategies. Thus, understanding the peculiarities of low-performing regions and the factors that promote low vaccination coverage is essential for planning public health policies with a view to achieving immunization goals^35^.

By analyzing the temporal and spatial distribution of polio vaccine coverage in the Brazilian states, the study showed significant heterogeneity in coverage between states and a strong downward trend in rates, especially in the Northeast region. Such gaps can facilitate the circulation of the poliovirus and put the susceptible population under threat.

The limitations of the study include the use of secondary data from a system that is subject to underreporting, either due to errors in data capture or incorrect completion.

Thus, it is also necessary to invest in health policies, such as information and vaccination campaigns that highlight, for parents and guardians of children, the benefits, safety and importance of vaccination, aiming to restore vaccination coverage at rates conducive to the protection of vulnerable populations against poliomyelitis, which is highly contagious and has high rates of morbidity and mortality.
