# ERRATUM

**DOI:** 10.1590/1980-549720230037erratum.1

**Published:** 2023-10-27

In the manuscript “Temporal and spatial distribution of polio vaccine coverage in Brazil between 1997 and 2021”, DOI: https://doi.org/10.1590/1980-549720230037, published in the Rev Bras Epidemiol. 2023; 26: e230037:

**Page 1, where it reads:**

Hévila Medeiros Ferreira Gomes Braga

**It should read:**

Hévila Ferreira Gomes Medeiros Braga

**Page 1, how to cite this article**

**Where it reads:**

Braga HMFG

**It should read:**

Braga HFGM

**Page 9, authors’ contributions**

**Where it reads:**

Braga HMFG

**It should read:**

Braga HFGM

